# A multicenter, randomized, and double-blind phase IV clinical trial to compare the efficacy and safety of fixed-dose combinations of amlodipine orotate/valsartan 5/160 mg versus valsartan/hydrochlorothiazide 160/12.5 mg in patients with essential hypertension uncontrolled by valsartan 160 mg monotherapy

**DOI:** 10.1097/MD.0000000000012329

**Published:** 2018-09-14

**Authors:** Youngkeun Ahn, Yongcheol Kim, Kiyuk Chang, Weon Kim, Moo-Yong Rhee, Kwang Soo Cha, Min Su Hyon, Chi Young Shim, Sung Yun Lee, Doo Il Kim, Sang Wook Kim, Sang-Wook Lim, Kyoo-Rok Han, Sang-Ho Jo, Nae-Hee Lee, Jun Kwan, Taehoon Ahn

**Affiliations:** a Department of Cardiovascular Medicine, Chonnam National University Hospital, Gwangju; b The Catholic University of Korea, Seoul St. Mary's Hospital; c Kyung Hee University Hospital, Seoul; d Dongguk University Ilsan Hospital, Goyang; e Pusan National University Hospital, Busan; f Soonchunhyang University Seoul Hospital, Seoul; g Severance Cardiovascular Hospital, Yonsei University College of Medicine, Seoul; h Inje University Ilsan Paik Hospital, Goyang; i Inje University Haeundae Paik Hostpital, Busan; j Chung-Ang University Hospital, Seoul; k CHA Bundang Medical Center, CHA University, Seongnam; l Hallym University Kangdong Sacred Heart Hospital, Seoul; m Hanllym University Sacred Heart Hospital, Anyang; n Soonchunhyang University Bucheon Hospital, Bucheon; o Inha University Hospital; p Heart Center, Gachon University Gil Hospital, Incheon, Republic of Korea.

**Keywords:** amlodipine orotate, fixed-dose combination, hypertension, valsartan

## Abstract

**Background:**

To determine whether the effectiveness and safety of fixed-dose combinations (FDCs) of amlodipine orotate/valsartan (AML/VAL) 5/160 mg are noninferior to those of valsartan/hydrochlorothiazide (VAL/HCTZ) 160/12.5 mg in hypertensive patients with inadequate response to valsartan 160 mg monotherapy.

**Methods:**

This 8-week, active-controlled, parallel-group, fixed-dose, multicenter, double-blind randomized controlled, and noninferiority trial was conducted at 17 cardiovascular centers in the Republic of Korea. Eligible patients had mean sitting diastolic blood pressure (msDBP) ≥90 mm Hg despite monotherapy with valsartan 160 mg for 4 weeks. Patients were randomly assigned to treatment with AML/VAL 5/160 mg FDC (AML/VAL) group or VAL/HCTZ 160/12.5 mg FDC (VAL/HCTZ) group once daily for 8 weeks. A total of 238 patients were enrolled (AML/VAL group, n = 121; VAL/HCTZ group, n = 117), of whom 228 completed the study.

**Results:**

At 8 weeks after randomization, msDBP was significantly decreased in both groups (−9.44 ± 0.69 mm Hg in the AML/VAL group and −7.47 ± 0.71 mm Hg in the VAL/HCTZ group, both *P* < .001 vs baseline). Between group difference was −1.96 ± 1.00 mm Hg, indicating that AML/VAL 5/160 mg FDC was not inferior to VAL/HCTZ 160/12.5 mg FDC at primary efficacy endpoint. Control rate of BP defined as the percentage of patients achieving mean sitting SBP (msSBP) <140 mm Hg or msDBP <90 mm Hg (target BP) from baseline to week 8 was significantly higher in the AML/VAL group than that in the VAL/HCTZ group (84.3% [n = 102] in the AML/VAL group vs 71.3% [n = 82] in the VAL/HCTZ group, *P* = .016). At 8 weeks after randomization, mean uric acid level was significantly increased in the VAL/HCTZ group compared to that at baseline (0.64 ± 0.08 mg/dL; *P* < .001). However, it was slightly decreased from baseline in the AML/VAL group (−0.12 ± 0.08 mg/dL; *P* = .085). The intergroup difference was significant (*P* < .001).

**Conclusion:**

The effectiveness and safety AML/VAL 5/160 mg FDC are noninferior to those of VAL/HCTZ 160/12.5 mg FDC in patients with hypertension inadequately controlled by valsartan 160 mg monotherapy.

## 1 Introduction

Hypertension is a leading cause of death and the most important risk factor for cardiovascular disease, cerebrovascular disease, and renal disease.^[1–3]^ Its prevalence is expected to increase from 972 million people in the year of 2000 to 1.56 billion by 2025.^[4]^ Korean National Health and Nutrition Examination Survey have revealed that age-standardized prevalence of hypertension is approximately 30% among adults over 30 years of age.^[5]^ In patients with hypertension, angiotensin-converting-enzyme inhibitors, angiotensin II receptor blockers (ARBs), calcium channel blockers (CCBs), and diuretics are all suitable for initial antihypertensive treatment.

Valsartan is a nonpeptide ARB used orally to treat hypertension. It inhibits angiotensin type II receptor involved in reducing aldosterone secretion.^[6]^ This causes arteriolar and venous dilation, resulting in a decrease in blood pressure (BP). Amlodipine is one of the most widely used long-acting dihydropyridine CCB, ensuring sustained antihypertensive effect over 24 hours after a single dose.^[7]^ Amlodipine orotate was developed based on amlodipine besylate. The BP reducing effect of amlodipine orotate treatment is similar to that of amlodipine besylate in patients with mild to moderate hypertension.^[8]^

However, antihypertensive monotherapy does not provide adequate BP control in two-thirds of patients.^[9]^ A combination of antihypertensive drugs is a useful and appropriate treatment option because it can be more effective in lowering BP than high-dose monotherapy in hypertensive patients unless BP control is achieved by monotherapy.^[10,11]^ Combination therapy chosen from angiotensin-converting-enzyme inhibitors, ARBs, CCBs, and diuretics is recommended first because it has shown relatively good results.^[12,13]^ However, direct comparison studies on which drugs are useful in combination therapy for hypertensive patients who are not effectively treated with ARB monotherapy are limited.

Therefore, the objective of this study was to determine whether the efficacy and safety of amlodipine orotate/valsartan (AML/VAL) 5/160 mg fixed-dose combination (FDC) were noninferior to those of valsartan/hydrochlorothiazide (VAL/HCTZ) 160/12.5 mg FDC for hypertensive patients with not quite effective response to valsartan 160 mg monotherapy.

## 2 Patients and methods

### 2.1 Study design and patients population

This 8-week, active-controlled, parallel-group, fixed-dose, multicenter, double-blind randomized controlled, and noninferiority trial was conducted at 17 cardiovascular centers in the Republic of Korea between March 2015 and November 2016. This study was approved by the Institutional Review Board of each participating study center (ClinicalTrials.gov registry number: NCT02433119). It was conducted in accordance with the Declaration of Helsinki and Good Clinical Practice guidelines. Written informed consent was obtained from all patients prior to participation.

List of the Institutional Review Board: Chonnam National University Hospital, Seoul St. Mary's Hospital, Kyung Hee University Hospital, Dongguk University Ilsan Hospital, Pusan National University Hospital, Soon Chun Hyang University Hospital Seoul, Severance Cardiovascular Hospital, Inje University Ilsan Paik Hospital, Inje University Haeundae Paik Hostpital, Chung-Ang University Hospital, CHA Bundang Medical Center, Hallym University Kangdong Sacred Heart Hospital, Hanllym University Sacred Heart Hospital, Soon Chun Hyang University Hospital Bucheon, Inha University Hospital, Gachon University Gil Hospital.

The present study included subjects aged ≥19 years who were diagnosed with essential hypertension at the first visit. Subjects with medical history or evidence of a secondary hypertension or history of hypersensitivity to CCB, ARB, or sulfonamide were excluded from this study. Additionally, subjects with severe hypertension defined as mean sitting systolic blood pressure (msSBP) ≥200 mm Hg or mean sitting diastolic blood pressure (msDBP) ≥120 mm Hg and those with difference in BP (msSBP ≥20 mm Hg or msDBP ≥10 mm Hg) between right and left arm at screening evaluation were excluded. The subjects were excluded from the study if they had uncontrolled diabetes (fasting glucose ≥200 mg/dL or HbA1c ≥9.0%), severe heart failure (New York Heart Association Functional class III, IV), ischemic heart disease (angina pectoris, myocardial infarction within 6 months), peripheral vascular disease, second and third degree atrioventricular block, past history of percutaneous tranluminal coronary angioplasty or coronary artery bypass surgery, clinically significant arrhythmia, past history of severe cerebrovascular event (within 6 months), transient ischemic attack (within 1 year), moderate to malignant retinopathy, abnormal renal function test result (creatinine clearance <30 mL/min or serum creatinine ≥2 mg/dL), abnormal liver function test result (aspartate transaminase and alanine transainase ≥3.0 times the upper limit of normal), hyperkalemia or hypokalemia, and past history of liver disease.

**Figure 1:**
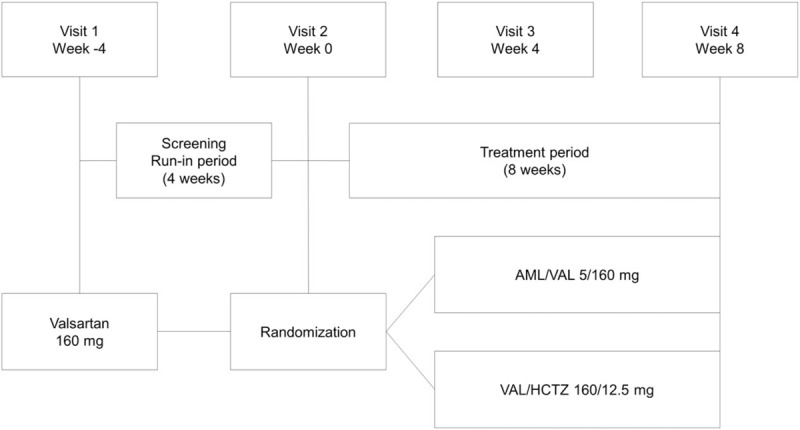
Study design. AML/VAL = amlodipine orotate/valsartan; VAL/HCTZ = valsartan/hydrochlorothiazide.

After screening, eligible patients who had msDBP ≥90 mm Hg despite monotherapy with valsartan 160 mg for 4 weeks were randomly assigned to receive either group AML/VAL 5/160 mg FDC (AML/VAL) or VAL/HCTZ 160/12.5 mg FDC (VAL/HCTZ) once daily for another 8 weeks without dose adjustment, using block-randomization (Fig. 1).

Patients were instructed to take the study drug once daily at the same time in the morning. At the screening visit, BP was measured in both arms 3 times and the arm with the higher average SiDBP was selected. At each visit, after at least 5 minutes of rest in a sitting position, siSBP, siDBP, and pulse rate were measured 3 times with a 2-minute interval between measurements in the same arm using a semi-automated sphygmomanometer (WatchBP Office TWIN200, Microlife AG Corporation, Widnau, Switzerland). The average of the 3 sitting BP measurements was used. Thereafter, adverse events (AEs), laboratory data, and physical examination were evaluated.

### 2.2 Randomization and masking

Randomization code was generated by a statistician using SAS (Ver. 9.3, SAS Institute, Cary, NC) and was stratified by center with a 1:1 ratio. The size of block was 2 or 4. When a patient was eligible for the study by the opinion of investigator, a subject screening number was provided at the visit 1, and subject enrollment number was provided when the subject satisfying enrollment criteria at the visit 2. Drugs corresponding to that the enrollment number were dispensed by a pharmacist. Both patients and investigators were blinded to the group assignment. In addition to the active medication, either AML/VAL or VAL/HCTZ, patients received identical matching placebo.

### 2.3 Efficacy evaluations

The primary efficacy endpoint was the reduction in msDBP from baseline to week 8. The secondary and other efficacy endpoints were to compare the following: reduction in msDBP from baseline to week 4, reductions in msSBP from baseline to weeks 4 and 8, control rate which was defined as the percentage of patients achieving msSBP <140 mm Hg or msDBP < 90 mm Hg (target BP) at week 8, response rate defined as the percentage of patients achieving msSBP ≥20 mm Hg and msDBP ≥10 mm Hg at week 8, and changes in laboratory parameters (total cholesterol, triglyceride, high-density lipoprotein cholesterol, low-density lipoprotein cholesterol, glucose, creatinine, and uric acid) at week 8.

### 2.4 Safety assessments

Safety assessments consisted of measuring vital signs, physical examinations, and recording all AEs and adverse drug reaction (ADRs) at each visit. Laboratory testing included routine hematological and biochemistry parameters at 8 weeks.

### 2.5 Statistical analysis

The primary efficacy endpoint was the change in msDBP from baseline to 8 weeks after treatment. A sample size of 115 in each group was estimated to be able to provide 80% power to noninferiority between AML/VAL 5/160 mg FDC and VAL/HCTZ 160/12.5 mg FDC at 2.5% significance level, assuming a standard deviation of 7.97 for AML/VAL 5/160 mg FDC and 8.22 for VAL/HCTZ 160/12.5 mg FDC with a noninferiority margin of 3 mm Hg. Considering drop-out rate at 10%, a total of 256 patients were enrolled to be randomized.

Efficacy analysis was based on full analysis set (consisted of all randomized patients who received at least 1 dose of study medication and who had at least 1 evaluable primary measurement) and per protocol (PP) set (consisted of all randomized patients who completed the study without any major protocol violation). Last-Observation-Carried-Forward approach was used to impute missing data for the full analysis set. Safety analyses included all randomized patients who received at least 1 dose of study drug and had a measurement of the safety endpoint.

For the primary endpoint, to demonstrate the noninferiority of AML/VAL 5/160 mg FDC to VAL/HCTZ 160/12.5 mg FDC, if the upper boundary of 2-sided 95% confidence interval (CI) for LS mean difference between the 2 groups was less than the pre-defined noninferiority margin of 3 mm Hg, AML/VAL 5/160 mg FDC was considered to be noninferior to VAL/HCTZ 160/12.5 mg FDC. An analysis of covariance (ANCOVA) model with baseline BP as a covariate was used to compare the primary endpoint and the secondary endpoints (msDBP reductions from baseline to week 4, msSBP reductions from baseline to weeks 4 and 8) between treatment groups. For the primary endpoint and the secondary endpoints (msDBP reductions from baseline to week 4, msSBP reductions from baseline to weeks 4 and 8), Chi-square test or Fisher exact test was used to compare control rates and response rates between the 2 groups.

For safety endpoints, frequencies and percentages of patients who experienced AEs were summarized and chi-square test or Fisher exact test was used for comparisons between treatment groups. For continuous variables, intergroup comparisons were performed using 2 sample *t*-test or Wilcoxon rank sum test.

Two-sided *P* values of less than .05 were considered to be statistically significant. All statistical analyses were conducted using SAS version 9.3 (SAS Institute, Cary, NC).

## 3 Results

### 3.1 Patient characteristics

**Figure 2:**
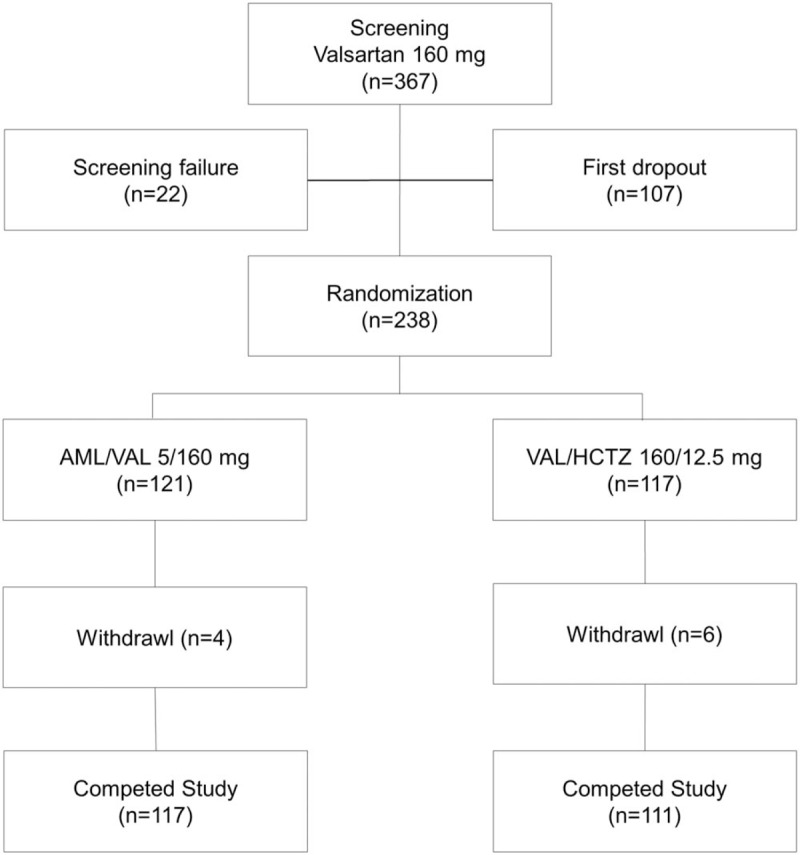
Flow chart of the patients through the study. AML/VAL = amlodipine orotate/valsartan; VAL/HCTZ = valsartan/hydrochlorothiazide.

Among 367 patients with hypertension after screening, a total of 238 eligible patients were randomly assigned to the AML/VAL group (n = 121) or the VAL/HCTZ group (n = 117), of whom 228 completed the study (Fig. 2). Baseline demographic and clinical characteristics of these patients are shown in Table 1.

**Table 1:**
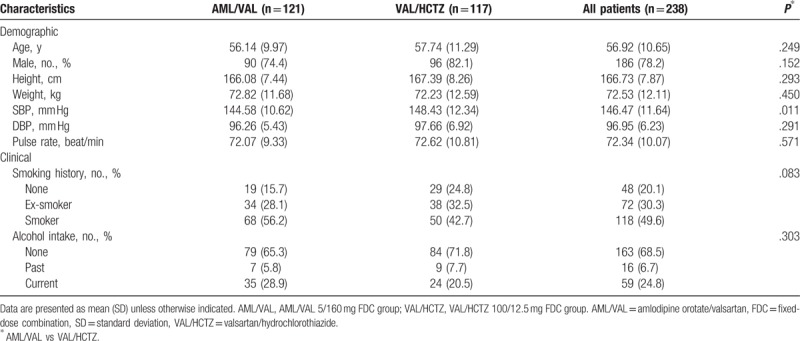
Baseline demographic and clinical characteristics of both groups.

### 3.2 Efficacy

**Figure 3:**
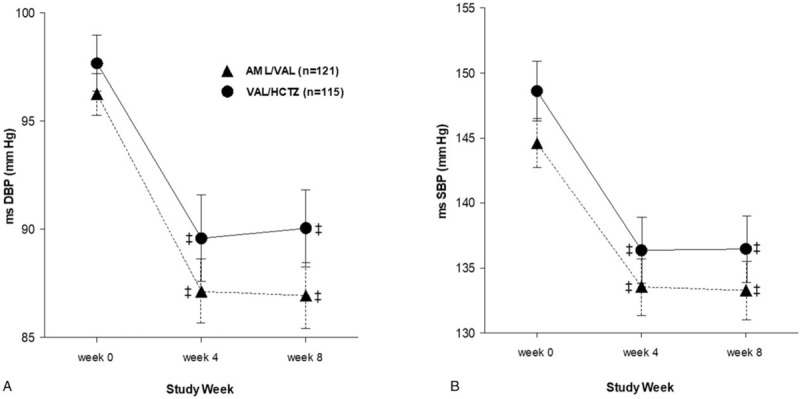
Mean blood pressure change from baseline through 8-week follow-up. (A) msDBP and (B) msSBP. AML/VAL, AML/VAL 5/160 mg FDC; VAL/HCTZ, VAL/HCTZ 160/12.5 mg FDC. ^‡^*P* < .001 versus baseline. AML/VAL = amlodipine orotate/valsartan, FDC = fixed-dose combination, msDBP = mean sitting diastolic blood pressure, msSBP = mean sitting systolic blood pressure, VAL/HCTZ = valsartan/hydrochlorothiazide.

Regarding primary efficacy endpoint, the reductions in msDBP from baseline to week 8 were −9.44 (0.69) mm Hg in the AML/VAL group and −7.47 (0.71) mm Hg in the VAL/HCTZ group (both *P* < .001 vs baseline) (Fig. 3A). The mean difference of the reduction in msDBP from baseline to 8 weeks was −1.96 mm Hg between the 2 groups (95% confidence interval [CI]: −3.93 to 0.00 mm Hg). The upper limit of 95% CI (at 0.00 mm Hg) was less than the predefined noninferiority margin (3 mm Hg), confirming that AML/VAL 5/160 mg FDC was not inferior to VAL/HCTZ 160/12.5 mg FDC. Similar results were obtained in the PP set. Reductions in msDBP after 8 weeks of study medication were −9.39 (0.70) mm Hg in the AML/VAL group and −7.42 (0.73) mm Hg in the VAL/HCTZ group (both *P* < 0.001 vs baseline). The mean difference of the reduction in msDBP from baseline to 8 weeks was −1.97 mm Hg between the 2 groups (95% CI: −3.98 to 0.03 mm Hg). The upper limit of 95% CI (at 0.03 mm Hg) was less than 3 mm Hg.

Regarding secondary efficacy endpoint, reductions in msDBP from baseline to week 4 were −9.23 (0.73) mm Hg in the AML/VAL group and −7.97 (0.74) mm Hg in the VAL/HCTZ group (both *P* < .001 vs baseline) (Fig. 3A). These msDBP reductions at week 4 were not significantly different between the 2 groups (*P* = .23). Reductions of msSBP from baseline to weeks 4 and 8 were −11.81 (0.99) mm Hg and −12.17 (1.05) mm Hg, respectively, in the AML/VAL group (both *P* < .001). They were −11.43 (1.01) mm Hg and −11.24 (1.08) mm Hg, respectively, in the VAL/HCTZ group (both *P* < .001) (Fig. 3B). These reductions in msSBP at week 4 or week 8 between the 2 groups were not significantly different (*P* = .786, *P* = .54, respectively). In PP analysis, reductions in msDBP from baseline to week 4 were −9.27 (0.73) mm Hg in the AML/VAL group (*P* < .001) and −8.12 (0.76) mm Hg in the VAL/HCTZ group (*P* < .001). These msDBP reductions at week 4 were not significantly different between the 2 groups (*P* = 0.23). Reductions of msSBP from baseline to week 4 and week 8 were −11.85 (0.96) mm Hg and −12.05 (1.06) mm Hg, respectively, in the AML/VAL group (both *P* < .001). They were −11.76 (1.00) mm Hg and −11.25 (1.10) mm Hg, respectively, in the VAL/HCTZ group (both *P* < .001). These reductions in msSBP at week 4 or week 8 between the 2 groups were not significantly different (*P* = .474, *P* = .604, respectively).

Control rate showed significant difference (84.3% in the AML/VAL group vs 71.3% in the VAL/HCTZ group, *P* = .016). However, response rate was not significantly different between the 2 groups (18.2% in the AML/VAL group vs 22.6% in the VAL/HCTZ group, *P* = .398) (Table 2). In PP analysis, response rate at week 8 was not significantly different between the 2 groups either (18.3% in the AML/VAL group vs 20.6% in the VAL/HCTZ group, *P* = .665).

**Table 2:**
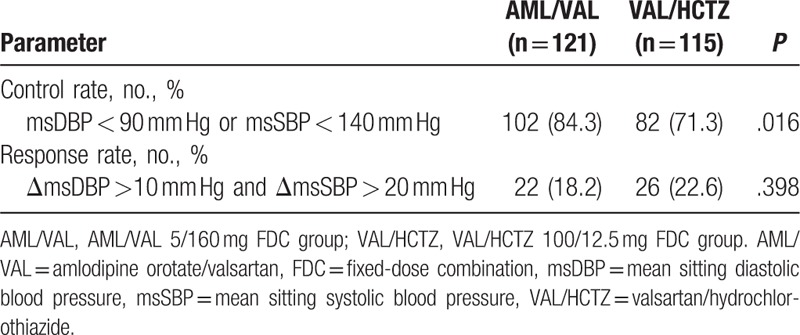
Control and Normalization rate and response rate by group.

**Figure 4:**
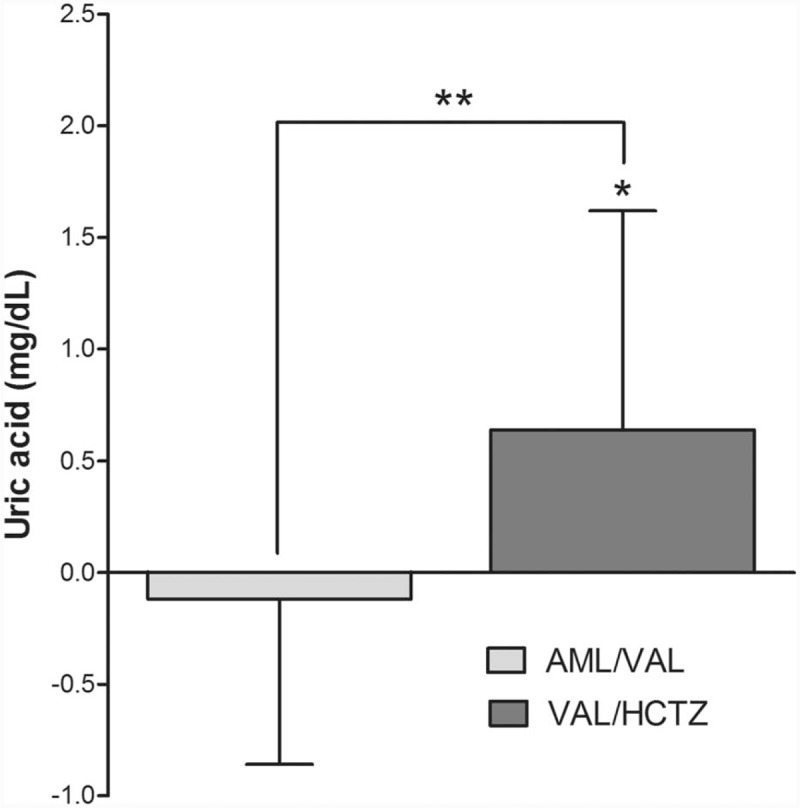
Uric acid level change after 8 weeks of medication. AML/VAL, AML/VAL 5/160 mg FDC; VAL/HCTZ, VAL/HCTZ 160/12.5 mg FDC. ∗*P* < .001 versus baseline. ∗∗*P* < .001 in intergroup difference. AML/VAL = amlodipine orotate/valsartan, FDC = fixed-dose combination, VAL/HCTZ = valsartan/hydrochlorothiazide.

Regarding changes of laboratory parameters at week 8, the mean uric acid level was significantly increased from baseline in the VAL/HCTZ group (0.64 [0.98] mg/dL, *P* < .001). However, it was slightly decreased from baseline in the AML/VAL group (−0.12 [0.08] mg/dL, *P* = .085). The intergroup difference was significant (*P* < .001) (Fig. 4). Lipid, glucose, or creatinine did not show significant changes in either group (Table 3)

**Table 3:**
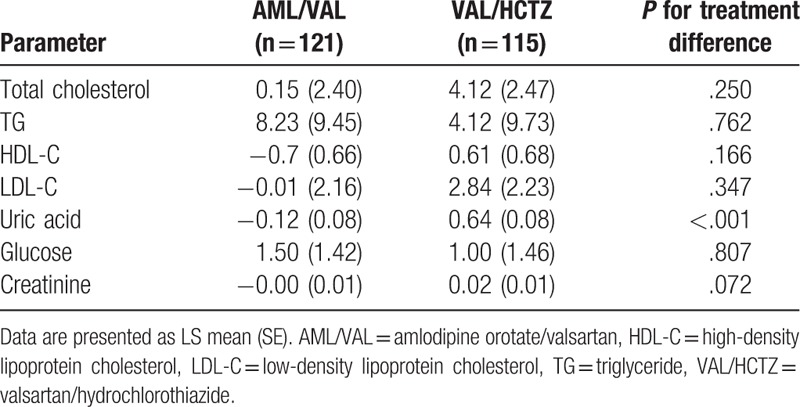
Changes of laboratory parameters at week 8 of both groups.

### 3.3 Safety

A total of 236 patients (121 in the AML/VAL group and 115 in the VAL/HCTZ group) were included in the safety set. There were no significant differences in incidence of overall AEs or ADRs between the 2 groups. No serious AEs or serious ADRs were found in either group (Table 4).

**Table 4:**
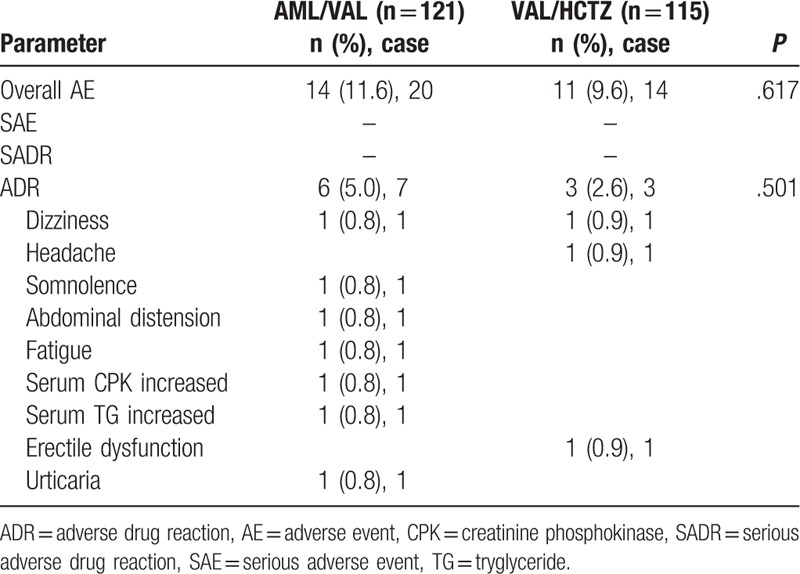
Summary of adverse events during the study period of both groups.

## 4 Discussion

Therapeutic efficacy of combination therapy has been well established. A meta-analysis of 42 randomized trials has shown a 5-fold reduction in BP with a combined use of 2 antihypertensive drugs of different classes compared to doubling the dose of 1 drug alone.^[14]^ Guidelines have recommended both combination therapies of ARB with an HCTZ and an ARB with a CCB to achieve target BP.^[15,16]^ In addition, FDC regimens have been well investigated to improve drug compliance in hypertension.^[17]^ Which ARB-based fixed combination therapy is useful for hypertension uncontrolled by ARB monotherapy remains unclear. Combination therapy with ARB and CCB is one of the preferred regimens. It is the most widely used 2-drug combination for BP reduction.^[15]^ AML/VAL FDC has clinically demonstrated significant BP lowering effect. It is also well-tolerated in several clinical trials.^[18,19]^ Furthermore, combination of fixed-dose amlodipine orotate and ARB has demonstrated the safety and biocompatibility of amlodipine besylate (first marketed as amlodipine) and ARB FDC.^[20]^

In our study, FDC of amlodipine orotate and valsartan 5/160 mg significantly decreased BP in patients with hypertension uncontrolled by valsartan 160 mg monotherapy. The DBP lowering effect of FDC of amlodipine orotate and valsartan 5/160 mg was not significantly different from that of FDC of valsartan and HCTZ 160/12.5 mg. In addition, FDC of amlodipine orotate and valsartan 5/160 mg provided rapid and significant BP reduction after only 4 weeks of medication. Although response rates did not differ significantly between the 2 groups, control rate (defined as achieving both target DBP or target SBP at msDBP <90 mm Hg and msSBP <140 mm Hg) at week 8 was significantly greater in the AML/VAL group compared to those in the VAL/HCTZ group. In terms of effectiveness, our data suggest that the FDC of amlodipine orotate and valsartan 5/160 mg has a rapid, significant BP lowering effect and high achievement rate of the target BP as well.

Hydrochlorothiazide selectively enhances urate reabsorption by acting as a counter-ion for urate transport.^[21]^ Valsartan does not have a molecular effect of uricosuric action through inhibition of uric acid transporter 1 in hypertensive patients when compared to losartan.^[22]^ Some studies have shown that valsartan can result in significant increase of serum uric acid level.^[23]^ In our study, uric acid level was significantly increased from baseline in the VAL/HCTZ group. However, FDC of amlodipine orotate and valsartan 5/160 mg did not increase serum uric acid level. Instead, it slightly decreased serum uric acid level. The cause of uric acid reduction in AML/VAL group might be due to amlodipine because amlodipine can significantly increase the clearance rate of uric acid.^[24]^ Furthermore, amlodipine orotate can decrease uric acid level as much as amlodipine besylate after 8 weeks of treatment^[8]^. Therefore, FDC of amlodipine orotate and valsartan 5/160 mg might be especially useful for hypertensive patients with hyperuricemia.

Our study has some limitations that are worth mentioning. First, the follow-up period was relatively short (only for 8 weeks). Second, it is difficult to generalize the results of this study because the population studied is different from the population treated in practice. Therefore, a large-scale study is needed to evaluate the long-term efficacy of FDC of amlodipine orotate and valsartan 5/160 mg.

## 5 Conclusions

The efficacy and safety of FDC with AML/VAL 5/160 mg were found to be noninferior to those of FDC with VAL/HCTZ 160/12.5 mg in patients with hypertension inadequately controlled by valsartan 160 mg monotherapy. In addition, treatment with FDC of AML/VAL 5/160 mg resulted in significantly higher control rate (achieving both target SBP and target DBP) compared to FDC of VAL/HCTZ 160/12.5 mg. In terms of safety, there were no serious AEs and serious ADRs in either group. FDC of AML/VAL 5/160 mg did not increase serum uric acid level either, although FDC of VAL/HCTZ 160/12.5 mg significantly increased serum uric acid level.

## Author contributions

All authors were involved in revising the original draft of the manuscript and approved the final draft. All authors were involved in the decision to submit this manuscript for publication. The research sponsor was involved in the design of the study, data collection, and data analysis.

**Conceptualization:** Doo Il Kim, Nae-Hee Lee, Jun Kwan.

**Funding acquisition:** Taehoon Ahn.

**Investigation:** Jun Kwan.

**Methodology:** Sang Wook Kim, Kyoo-Rok Han, Jun Kwan.

**Supervision:** Sung Yun Lee, Sang-Wook Lim, Kyoo-Rok Han, Sang-Ho Jo, Taehoon Ahn.

**Validation:** Sang Wook Kim, Nae-Hee Lee, Taehoon Ahn.

**Writing – original draft:** Youngkeun Ahn, Yongcheol Kim.

**Writing – review & editing:** Kiyuk Chang, Weon Kim, Moo-Yong Rhee, Kwang Soo Cha, Min Su Hyon, Chi Young Shim, Sang-Wook Lim, Kyoo-Rok Han, Sang-Ho Jo, Nae-Hee Lee.
